# PlGF and VEGF-A Regulate Growth of High-Risk MYCN-Single Copy Neuroblastoma Xenografts via Different Mechanisms

**DOI:** 10.3390/ijms17101613

**Published:** 2016-09-23

**Authors:** Karin Zins, Daniel Kovatchki, Trevor Lucas, Dietmar Abraham

**Affiliations:** 1 Division of Cell and Developmental Biology, Center for Anatomy and Cell Biology, Medical University of Vienna, A-1090 Vienna, Austria; karin.zins@meduniwien.ac.at (K.Z.); zardinax@gmail.com (D.K.); trevor.lucas@meduniwien.ac.at (T.L.); 2 Comprehensive Cancer Center (CCC), Medical University of Vienna, A-1090 Vienna, Austria

**Keywords:** placental growth factor, vascular endothelial growth factor A, vascular endothelial growth factor receptor 2, neuroblastoma, xenograft model

## Abstract

Neuroblastoma (NB) is the most common extracranial solid tumor of childhood and is a rapidly growing, highly-vascularized cancer. NBs frequently express angiogenic factors and high tumor angiogenesis has been associated with poor outcomes. Placental growth factor (PlGF) is an angiogenic protein belonging to the vascular endothelial growth factor (VEGF) family and is up-regulated mainly in pathologic conditions. Recently, PlGF was identified as a member of a gene expression signature characterizing highly malignant NB stem cells drawing attention as a potential therapeutic target in NB. In the present study, we sought to investigate the expression of PlGF in NB patients and the effect of PlGF inhibition on high-risk *MYCN*-non-amplified SK-N-AS NB xenografts. Human SK-N-AS cells, which are poorly differentiated and express PlGF and VEGF-A, were implanted subcutaneously in athymic nude mice. Treatment was done by intratumoral injection of replication-incompetent adenoviruses (Ad) expressing PlGF- or VEGF-specific short hairpin (sh)RNA, or soluble (s)VEGF receptor 2 (VEGFR2). The effect on tumor growth and angiogenesis was analyzed. High PlGF expression levels were observed in human advanced-stage NBs. Down-regulating PlGF significantly reduced NB growth in established NB xenografts by reducing cancer cell proliferation but did not suppress angiogenesis. In contrast, blocking VEGF by administration of Ad(sh)VEGF and Ad(s)VEGFR2 reduced tumor growth associated with decreased tumor vasculature. These findings suggest that PlGF and VEGF-A modulate *MYCN*-non-amplified NB tumors by different mechanisms and support a role for PlGF in NB biology.

## 1. Introduction

Neuroblastoma (NB) arises from primitive neuroepithelial cells of the neural crest and is the most frequently occurring solid tumor in children. Although some NB patients have a good response to treatment, a subset of NB patients is poorly responsive to current therapeutic schemes [1]. Consequently, NBs are heterogeneous in their biological characteristics and tumor stage, as well as patient age, are important prognostic factors that strongly correlate with survival and are used for treatment assignment [2]. Genetic abnormalities, including DNA ploidy and amplification of the *MYCN* oncogene, which is observed in about 20% of neuroblastoma cases, also play a role in determining the tumor phenotype and predicting the outcome. Together with other prognostic factors, they have been used to categorize patients in four categories, very low risk, low risk, intermediate risk, and high risk [1,3].

Additionally, several reports on NB have shown the important dependency of NB on angiogenesis, demonstrating that high vascularity is characteristic for the progressed tumor stages and poor outcome in human NB [4,5]. High-risk NB patients have poor prognosis and a very unfavorable balance of the regulators with several pro-angiogenetic factors working together to achieve more effective angiogenesis and aggressive tumor growth [6]. Thus, inhibition of angiogenesis has been considered as a strategy for therapy of NB [6].

Increased expression of VEGF-A, a central mediator of tumor angiogenesis, was found in advanced-stage NBs (stages 3 and 4) compared with low-stage tumors (stages 1, 2, and 4S) [7]. It has been reported that *MYCN* up-regulates VEGF-A in NB cells [8] and *MYCN*-amplified NBs induced a higher angiogenic response compared to the non-amplified ones [9]. However, results from *MYCN*-amplified NB xenografts suggest that established *MYCN*-amplified NB tumors are relatively VEGF-independent, and display the ability to up-regulate alternative pro-angiogenic mechanisms, including the PlGF pathway, that may stabilize vasculature and reduce the efficacy of an antiangiogenic therapy [10].

Recently, PlGF-2 was identified as gene belonging to a gene expression signature elevated in highly malignant NB stem cell lines [11]. PlGF is a pleiotropic factor that activates VEGFR1 (also named FLT-1), but not VEGFR2 (FLK-1/KDR), and affects different cell types and regulates various biological responses, including vessel growth and maturation [12]. The involvement of PlGF in stimulating angiogenesis depends on direct effects on vascular cells (endothelial cells and pericytes) as well as nonvascular cells, such as tumor cells and macrophages [13]. Activation and recruitment of macrophages by PlGF [14] results in tumor infiltration and production of pro-angiogenic factors by these tumor-associated macrophages to promote tumor growth [15]. PlGF has a redundant role in health, but has a non-redundant role in disease states such as inflammation and cancer. Moreover, PlGF overexpression correlated with tumor vascularity and was associated with poor outcome in different types of cancers [16]. However, PlGF inhibition revealed conflicting results, documenting antitumor efficacy by blocking PlGF in some, but not all studies [13].

In the present study, we analyzed the expression PlGF in NB patients. We also aimed to define the potential anti-tumor and antiangiogenic effects of an anti-PlGF treatment in comparison to anti-VEGF treatments in a xenograft model of *MYCN*-nonamplified NB.

## 2. Results

### 2.1. Placental Growth Factor (PlGF) Expression Is up-Rregulated in Neuroblastoma

We first examined serum PlGF levels of 37 children with NB and evaluated PlGF mRNA and protein expression in NB biopsies by quantitative real-time reverse transcriptase–polymerase chain reaction (qRT-PCR) and Western blotting. Patient characteristics, including tumor staging and location, are detailed in Table 1. Most of the tumors were located in the adrenal with clinical stages I–IV.

**Figure 1:**
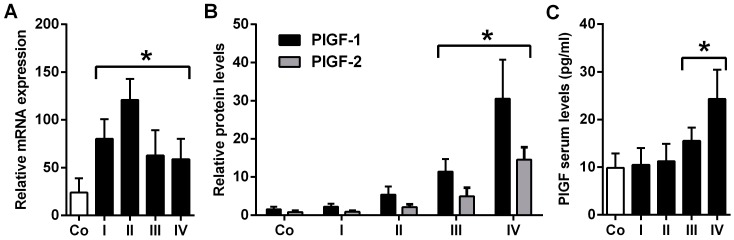
Placental growth factor (PlGF) expression in neuroblastoma (NB) and control biopsies. (**A**) qRT-PCR detecting PlGF-1 and PlGF-2. PlGF mRNA expression is significantly elevated in all tumor stages vs. controls; (**B**) PlGF protein expression is up-regulated in NB stages III and IV; and (**C**) PlGF serum levels are increased in tumor stages III and IV. Data are mean ± SD. Asterisks (*) indicate *p* < 0.04 vs. controls (Co).

PlGF (PlGF-1 and PlGF-2) mRNA expression was significantly up-regulated in biopsies of stage I–IV NBs vs. control biopsies (*p* < 0.01; Figure 1A). PlGF-1 and PlGF-2 protein levels were significantly up-regulated in NB stages III–IV, but not stage I and II, as compared to control biopsies (*p* < 0.003; Figure 1B). Analysis of serum PlGF levels revealed significantly increased serum levels in stages III (*p* = 0.03) and IV (*p* < 0.001) compared to sera from control patients (Figure 1C). These data show a significant expression of PlGF in NB patients supporting a role for PlGF in NB.

### 2.2. Generation of Replication-Incompetent Adenoviruses (Ads) Expressing Short Hairpin (shRNA) Specific to PlGF and VEGF and of Soluble (s)VEGFR2-Expressing Replication-Incompetent Ads

In humans, four PlGF isoforms have been described, whereas mice only express the equivalent of PlGF-2 [13]. Likewise, VEGF-A exists in different isoforms, which are generated by alternative splicing from a single VEGF pre-mRNA [17]. To generate interfering RNAs that will degrade all PlGF and VEGF isoforms, we designed siRNA sequences that were within PlGF exon 7 and VEGF_121/120_ exon 1 mRNA.

**Figure 2:**
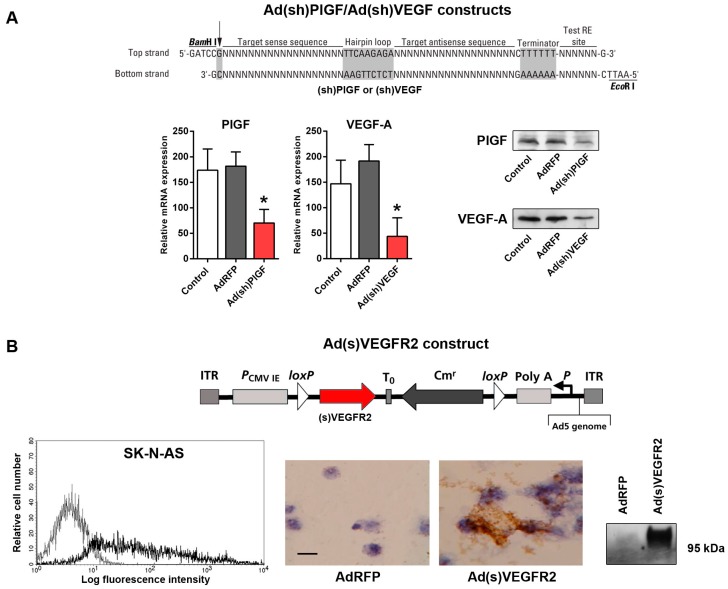
Design and characterization of Ad(sh)PlGF, Ad(sh)VEGF, and Ad(s)VEGFR2. (**A**) Design of PlGF and VEGF-A-specific shRNA constructs. Cells were transduced for 48 h with Ad(sh)PlGF or Ad(sh)VEGF, and the knockdown of endogenous mRNA and protein expression was measured by qRT-PCR and Western blotting for PlGF and VEGF-A. Graphs show shRNA-mediated in vitro knockdown of PlGF and VEGF-A gene expression in SK-N-AS cells. Western blots of cell lysates from SK-N-AS cells transduced with Ad(sh)PlGF or Ad(sh)VEGF show suppressed target gene expression. Each value represents the mean ± SD of three independent experiments. Arrow indicates the purine residue required for RNA Pol III to initiate transcription. Grey color denotes the hairpin loop sequence used to generate shRNAs and the termination poly(T) tract. Asterisks (*) indicate *p* < 0.03 vs. controls; (**B**) **Upper panel**: design of the Ad(s)VEGFR2 construct; **Lower panel**: shows FACS analysis of SK-N-AS cultures 48 h after infection with AdRFP showing transduction of SK-N-AS cells. Data show representative histograms of 10^4^ gated events (**left**). Immunohistochemical detection of (s)VEGFR2 on cytospins of SK-N-AS cells infected with AdRFP or Ad(s)VEGFR2; scale bar represents 10 µm (**middle**). Secretion of (s)VEGFR2 by SK-N-AS cells. VEGFR2 Western blot of culture supernatants from SK-N-AS cells transduced with Ad(s)VEGFR2 and control AdRFP (**right**).

PlGF and VEGF-A-specific shRNA-expressing plasmids were then constructed to express shRNAs under the control of a human U6 promoter. The shRNA oligonucleotides containing the 19-nucleotide PlGF or VEGF-specific targeting sequences were cloned into RNAi-Ready pSIREN-plasmid, generating PlGF and VEGF-specific shRNA-expressing plasmids p(sh)PlGF and p(sh)VEGF-A (Figure 2A). We then proceeded to construct E1/E3-deleted replication-incompetent Ads expressing shPlGF and shVEGF. To determine the effect of shRNA expression on PlGF and VEGF mRNA and protein levels, SK-N-AS cells were transduced with Ad(sh)PlGF or Ad(sh)VEGF. Transduction with AdRFP served as control for determining potential Ad-related effects. Two days following transduction, qRT-PCR and Western blotting were performed to determine the level of endogenous PlGF and VEGF mRNA and protein expression in SK-N-AS cells. Both, PlGF and VEGF mRNA and protein levels significantly decreased in cells transduced with Ad(sh)PlGF (*p* < 0.02) and Ad(sh)VEGF (*p* < 0.025), respectively, as shown in Figure 2A. In contrast, treatment of cells with AdRFP was ineffective. We next constructed an E1/E3-deleted replication-incompetent Ad-expressing soluble VEGFR2 acting as decoy receptor for VEGF (Figure 2B). First, we determined the rates of infection for the NB cells in vitro. Fluorescence-activated cell sorting (FACS) analyses of SK-N-AS cell cultures transduced with AdRFP showed a mean 52% rate of infection with AdRFP (Figure 2B). To determine expression and secretion of soluble VEGFR2 protein, SK-N-AS cancer cells were transduced with Ad(s)VEGFR2 and AdRFP as control. Two days following transduction, immunohistochemical analysis of cytospin slides demonstrated that (s)VEGFR2 was expressed in Ad(s)VEGFR2-transduced SK-N-AS cells, but not AdRFP transduced controls (Figure 2B). Western blotting analysis of culture supernatants of SK-N-AS cells transduced with Ad(s)VEGFR2 showed the presence of secreted soluble VEGFR2 protein compared to cells infected with AdRFP (Figure 2B).

Taken together, these in vitro data show robust adenoviral infection of SK-N-AS cells and efficient shRNA target gene suppression as well as efficient secretion of (s)VEGFR2 from Ads-transduced NB cells.

### 2.3. PlGF Blockade Reduces Growth of MYCN-Non-Amplified NB Xenografts

**Figure 3:**
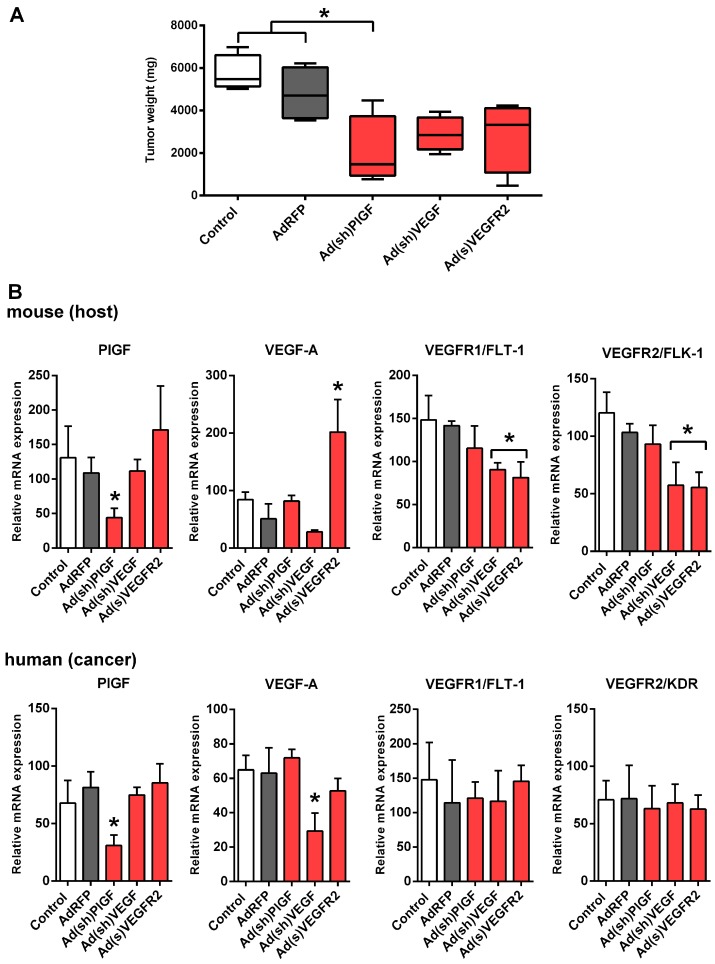
PlGF knockdown reduces growth of SK-N-AS xenografts. NB cells were injected subcutaneously into athymic nude mice and tumor growth and gene expression were analyzed. (**A**) Quantification of tumor weight of SK-N-AS xenografts on day 22 from mice treated with Ad(sh)PlGF, Ad(sh)VEGF, Ad(s)VEGFR2, or AdRFP and PBS as controls (*n* = 4 per group). Box and whisker plots show the mean, quartiles and tenth and ninetieth percentiles of the data. PlGF and VEGF blockade suppressed tumor weight of NB xenografts in mice. Data are mean ± SD. *, significantly different from control tumors; (**B**) mRNA expression of PlGF, VEGF, and VEGF receptors of SK-N-AS NB tumor tissue on day 22. Graphs show the results of qRT-PCR for murine (host) and human (cancer cell-derived) PlGF, VEGF-A, VEGFR1, and VEGFR2. Data are mean ± SD. *, significantly different from control tumors.

To assess the role of PlGF on SK-N-AS tumor growth, we used a xenograft model in which SK-N-AS cells were injected subcutaneously into mice. Ad(sh)PlGF, Ad(sh)VEGF, or Ad(s)VEGFR2 were injected into established tumors on day 10 and the effects on tumor weight were compared. AdRFP and PBS were used as controls. At the beginning of treatment on day 10 after tumor cell inoculation, mice developed human tumors of comparable size as measured using a caliper. On day 22, at which time the animals were sacrificed, the mean tumor mass of the Ad(sh)PlGF treated group (2045 ± 1654 mg) was significantly reduced compared to PBS treated control (5738 ± 853 mg) and AdRFP (4791 ± 1266 mg) control mice (*p* < 0.032; Figure 3A). Tumor masses in Ad(sh)VEGF (2897 ± 819 mg) and Ad(s)VEGFR2 (2838 ± 1675 mg) treated groups were also reduced compared with the PBS and AdRFP control groups, although not reaching statistical significance (Figure 3A). There were no significant differences in mean body weights between groups (data not shown).

### 2.4. PlGF Gene Silencing Does Not Affect mRNA Levels of Its Receptor VEGFR1/FLT-1 in NB Xenografts

Both, host (murine) and cancer cell (human) tissue PlGF mRNA levels significantly decreased in NB xenografts (*p* < 0.034) along with the tumor suppression following Ad(sh)PlGF treatment as compared to controls (Figure 3B). No significant differences were observed for PlGF levels in SK-N-AS xenografts following Ad(sh)VEGF and Ad(s)VEGFR2 treatment.

Likewise, cancer cell-derived VEGF-A mRNA levels significantly decreased in NB xenografts (*p* < 0.003) following Ad(sh)VEGF treatment (Figure 3B). Host VEGF mRNA levels were also reduced, although not reaching statistical significance. In contrast, mRNA levels of host, but not cancer cell-derived VEGF-A, were significantly up-regulated following Ad(s)VEGFR2 treatment (*p* < 0.001), while Ad(sh)PlGF treatment did not affect VEGF-A levels (Figure 3B).

Host VEGFR1 and VEGFR2 mRNA levels were significantly down-regulated following Ad(sh)VEGF and Ad(s)VEGFR2 treatment in SK-N-AS xenografts (*p* < 0.008 and *p* < 0.002, respectively), while cancer cell VEGFR1 and VEGFR2 were not significantly different from controls. No significant differences were observed for VEGFR1 and VEGFR2 mRNA levels following Ad(sh)PlGF treatment (Figure 3B).

These data suggest that the blockade of PlGF does not affect gene expression of its receptor VEGFR1; in contrast, VEGF-blockade affects gene expression of host VEGFR1 and VEGFR2 in SK-N-AS tumors in vivo.

### 2.5. PlGF and VEGF Blockade Differentially Change the Histologic Appearance of NB Xenografts

**Figure 4:**
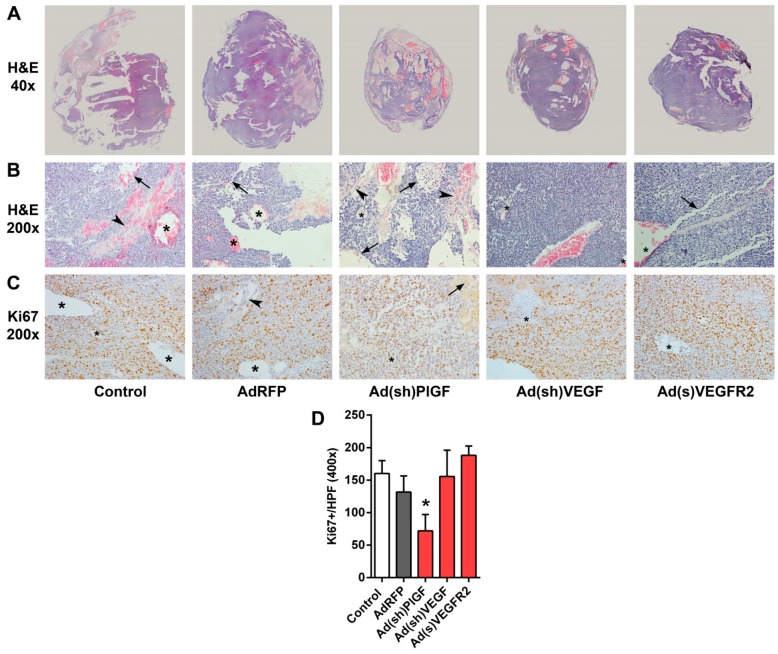
Representative histological sections of SK-N-AS tumors. (**A**) H and E-stained cross-sections of SK-N-AS NB tissue specimens demonstrated large hemorrhagic areas, which were less abundant in Ad(sh)VEGF and Ad(s)VEGFR2-treated tumors. Original magnifications, ×40; (**B**) H and E-stained sections of SK-N-AS tumors. Original magnifications, 200×. Arrowheads in B and D point to vascular tufts frequently seen in control, AdRFP, and Ad(sh)PlGF groups. Arrows point to hemorrhagic or potential areas of vascular mimicry. Asterisks label vessels lined by endothelial cells. Ad(sh)VEGF and Ad(s)VEGFR2 groups display more dense structures; (**C**) representative images and (**D**) quantification of Ki-67-immunostained tumor cell sections. Original magnifications, 200×. The mean number of Ki-67-positive cells (brown cells) per 10 high power fields (HPFs) of the treated tumors and controls are shown in the bar graph. Significantly less Ki-67-positive cancer cells were present in Ad(sh)PlGF-treated tumors. Data are mean ± SD. *, significantly different from control tumors.

Cross-sections of H and E-stained tissue specimens showed tumors with a variable appearance. Untreated and AdRFP control tumors were bulky with hemorrhagic areas. Though smaller in size, Ad(sh)PlGF group also appeared as extensively hemorrhagic tumors. In contrast, Ad(sh)VEGF and Ad(s)VEGFR2 groups were rather firm, with only limited areas of hemorrhage (Figure 4A). In Figure 4B, representative H and E-stained sections at higher magnification (200×) demonstrate the morphological changes, including changes of the vascular pattern observed in Ad(sh)VEGF and Ad(s)VEGFR2 groups as compared to control groups. Control, AdRFP, and Ad(sh)PlGF groups contained irregular vascular structures defined as multiluminal vascular tufts containing vessels with multiple lumens and thickened walls besides normal, thin-walled vessels. In addition, hemorrhagic or potential areas of vascular mimicry, i.e., vessel-like structures lined by tumor cells, appeared in these groups.

Treatment with Ad(sh)PlGF shrinks the tumor. Accompanying the reduction in tumor growth, Ki67^+^ proliferating NB cells in SK-N-AS xenografts were significantly reduced in the PlGF-shRNA group by 55% as compared to controls (*p* < 0.001; Figure 4C,D). In contrast, although treatment with Ad(sh)VEGF and Ad(s)VEGFR2 also reduced tumor growth, it makes the tumor firmer, associated with reduced hemorrhaging, but does not affect cancer cell proliferation.

**Figure 5:**
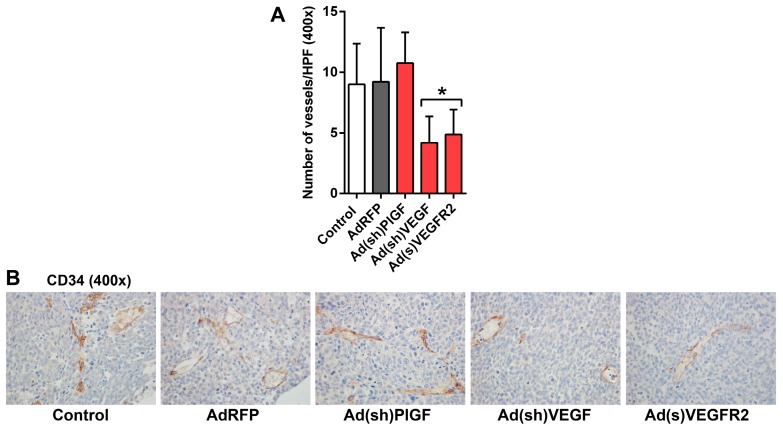
Endothelial microvessels in neuroblastoma xenografts. (**A**) Quantification of CD34-immunostained tumor endothelial microvessels per field and (**B**) representative images of CD34-stained tumor cell sections. Nuclei are stained with DAPI (blue). Original magnifications, 400×. The mean number of CD34-stained microvessels (brown cells) per HPF of the treated tumors and controls are shown in the bar graph. Significantly fewer endothelial microvessels were present in Ad(s)VEGFR2- and Ad(sh)VEGF-treated tumors. Data are mean ± SD. *, significantly different from control tumors.

In order to investigate the effect on tumor microvessels, endothelial cells lining endothelial microvessels (EM) were detected by staining with an anti-CD34 monoclonal antibody. In untreated and AdRFP control tumors, mean overall EM density was 9.0 and 9.2 microvessels per 40× field visually (range 5–15 and 3–19), respectively. In Ad(sh)PlGF-treated tumors the mean overall EM density was 10.8 microvessels per 40× field visually (range 6–14), while in Ad(s)VEGFR2- and Ad(sh)VEGF-treated tumors the mean overall EM density was 4.2 and 4.9 microvessels per 40× field visually (range 1–9 in both groups), respectively. Thus, a significantly lower microvessel density was only encountered in the Ad(s)VEGFR2 and Ad(sh)VEGF groups compared to control groups (*p* < 0.022) (Figure 5A,B). These data demonstrated that EM formation in tumors is not affected by PlGF blockade in neuroblastoma xenografts.

Taken together, PlGF and VEGF-blockade resulted in a heterogeneous histological appearance of SK-N-AS tumors. PlGF-blockade reduced tumor cell proliferation but did not affect endothelial microvessel density, while VEGF-blockade reduced microvessel density and induced some degree of vessel normalization in *MYCN*-unamplified SK-N-AS NBs.

## 3. Discussion

The very poor prognosis of advanced NB and the modest therapeutic achievements underline the need for designing alternative therapeutic strategies, such as adding an anti-angiogenesis treatment. Angiogenesis is a hallmark of tumor pathogenesis and contributes to the progression of cancer and is a logical target for new and established treatment strategies [18]. It has become clear that there are many possible ways to suppress angiogenesis, but there are even more ways for the tumor to evade this suppression, especially in strongly vascularized and highly malignant tumors like NB. NB represents a real challenge for antiangiogenic therapies, since NB tumors have a very early sprouting vasculature and build metastasis early. Although they seem to follow the common angiogenesis pathways, NB has become a real challenge and a measure of success for most antiangiogenic therapies through the many mechanisms used by the tumor to ensure its blood supply [19,20]. Mechanisms that have been described in the formation of NB tumor vasculature include sprouting of new blood microvessels from pre-existing capillaries under the influence of VEGF-A [4]. VEGF-A binds to the receptor tyrosine kinases (RTK) VEGFR1 (FLT-1) and VEGFR2 (FLK-1, KDR), the latter being the major mediator of the angiogenic effects of VEGF-A. Moreover, the VEGF family and its receptors are major mediators of tumor angiogenesis and targets for antiangiogenic therapies [18]. Increased expression of VEGF-A has been described in advanced NBs (stages III and IV) [7,21,22] and NB progression correlated with increased levels of VEGF and high tumor vascularization [9]. In addition, Kang et al. reported that *MYCN* upregulates VEGF-A in NB cells [8]. This finding is in line with the induction of a higher angiogenic response in *MYCN*-amplified NBs compared to the non-amplified ones [9]. Additionally, increased expression of VEGF-A and -D, as well as reduced expression of the inhibitors VEGFR1 and sVEGFR2, a naturally occurring soluble form of VEGFR2, was observed in *MYCN*-amplified stage 4 NB [23]. However, there are results that challenge the unequivocal functions of VEGFs for the progression of NB. Vessel density was not predictive of survival in a cohort of NB patients [24]. In addition, some authors have emphasized the redundancy in angiogenic factor expression [7] and the heterogeneity of angiogenesis stimulators and inhibitors in NB [25,26]. Consistently, VEGF blockade rapidly elicits alternative proangiogenic pathways in NB [10].

Thus, it is not surprising that clinical results of VEGF inhibitors have only demonstrated limited responses in NB [27,28]. In general, clinical outcome of antiangiogenic treatment is associated with the development of resistance in a substantial fraction of tumors and the increased risk of invasion and metastasis [29]. Moreover, VEGF-A maintains the quiescent endothelial cells of healthy vessels, and, therefore, antiangiogenic therapies cause grave side effects, including bleeding and disturbed wound healing, that can lead to life-threatening conditions in cancer patients [30].

An attractive alternative for overcoming these problems is the modulation of PlGF/VEGFR1 signaling, since PlGF expression, unlike that of VEGF, is undetectable in most organs in healthy conditions, but is highly up-regulated in tumor conditions [31]. Thus, it has been suggested that PlGF blockade might inhibit disease processes without affecting normal health. [13]. In addition, it has been suggested that PlGF/VEGFR1 signaling may play a role in the development of resistance to inhibition of VEGF signaling in *MYCN*-amplified NB xenografts [10].

In this study, we demonstrate that PlGF is up-regulated in NB tissue and serum of patients and provide experimental evidence for a cancer promoting activity of PlGF in *MYCN*-non-amplified NB. Intriguingly, we demonstrated that PlGF inhibition reduced the proliferation of tumor cells in established *MYCN*-non-amplified NB xenografts resulting in suppression of tumor growth, but unexpectedly did not alter the tumor vasculature. In contrast, VEGF blockade affected the tumor vasculature but did not affect tumor cell proliferation. These results suggest that both PlGF and VEGF are critical to NB growth in established *MYCN*-non-amplified NB and that they act via different mechanisms. Interestingly, PlGF mRNA levels were significantly up-regulated in all tumor stages, being most pronounced in stage II tumors, a pattern opposite to that of PlGF protein and serum levels, which were highest in advanced stages III and IV. This discrepancy can be explained, at least in part, by post-transcriptional regulation of PlGF mRNA under pathological conditions. In support of this, up-regulation of PlGF protein without a concomitant increase in PlGF mRNA was shown in a retinopathy mouse model [32]. Another report described extension of PlGF mRNA half-life as a post-transcriptional effect in coronary artery smooth muscle cells [33]. In this context, it should be noted that we did not investigate the PlGF-4 isoform, since this isoform has only been described in normal human trophoblast and human umbilical vein endothelial cells (HUVECs) [34]. Thus, PlGF-4 is not likely to play a role in neuroblastoma.

PlGF transmits its signal through FLT-1 and affects a wide range of different cell types and induces various biological effects, such as vessel growth and maturation [13]. In tumors, PlGF is not only produced by malignant cells, but also by most types of stromal cells and recent studies document a tumor cell-stroma crosstalk, in which tumor cells can “educate” stroma cells to produce PlGF [13]. In our model, murine and human PlGF was present in NB tissue, supporting this hypothesis for NB. It is known that PlGF can promote tumor growth via various distinct mechanisms, including stimulation of vessel growth and maturation and PlGF blockade can promote vessel normalization [13]. However, in our NB model, PlGF blockade did neither reduce vessel tortuosity nor improve vessel patency or normalize sinusoidal capillarization as described in other tumor models [35]. An explanation might be that reduced PlGF levels may favor the formation of angiogenic VEGF homodimers that are known to promote vessel disorganization [13]. Together with our observation of FLT-1 expression by SK-N-AS cells and down-regulated tumor cell proliferation following PlGF inhibition, these findings suggest that the PlGF blockade directly inhibits NB cell growth rather than affecting vessel organization. Moreover, endothelial microvessel density was not affected by PlGF blockade. In support of this, Bais et al. [36] showed that PlGF blockade did not inhibit tumor angiogenesis during primary tumor growth in several models but inhibited metastasis and primary tumor growth. Another study confirmed that PlGF blockade slowed down chronic myeloid leukemia progression in part by preventing direct growth stimulatory effects of PlGF on leukemia cells [37]. However, the same study also found that PlGF signaling in the stromal compartment contributed to leukemia progression. Thus, we cannot rule out that additional effects of PlGF on stromal cells contribute to NB growth.

Furthermore, experimental models using human melanoma and sarcoma xenografts in mice showed elevated murine but not human PlGF levels following anti-VEGF treatment, suggesting that PlGF up-regulation by VEGF blockade is at least in part a host response [38]. Partly consistent herewith, we observed a moderate up-regulation of host-derived murine PlGF mRNA in our NB xenograft model in the response of the tumor to VEGF-A inhibition by Ad(s)VEGFR2. This effect, however, seems to be context-dependent, as Ad(sh)VEGF-A treatment did not result in an up-regulation of PlGF mRNA. Nevertheless, the inhibition of PlGF could provide an attractive means of supplementing an anti-VEGF therapy. This hypothesis is also supported by findings in this study, which show that SK-N-AS NBs differently react to the inhibition of PlGF and VEGF-A. Ad(sh)VEGF and Ad(s)VEGFR2 treatment caused remarkable histological changes, such as reduced hemorrhage and vascular density, what looks like vessel normalization, which has been described for VEGF blockade [39]. In contrast, in PlGF-treated NB xenografts we observed pathological vascular patterns, especially angiogenesis with a distorted architecture and an irregular lining of discontinuous endothelial cells, which was not different from controls. In addition, tufts of endothelial and perithelial cells and glomeruloid structures, which forces the capillary network to transform focally into irregular structures, similar to findings in NB patient tissue of NB Schwannian stroma-poor tumors [40], were found in both PlGF-treated and control groups. However, our data also indicate that targeting of a single angiogenic molecule or pathway, i.e., PlGF or VEGF, is insufficient to completely suppress tumor growth. NB often resists angiogenesis suppression and often develops resistance to anti-VEGF treatments through multiple mechanisms [29]. In addition to sprouting under the influence of VEGF-A and other proangiogenic growth factors [7,41], alternative modes of vessel formation occur in NB, such as vascular mimicry, in which tumor cells form vascular channels in vivo and in vitro [42,43]. Of interest, it has been suggested that vascular mimicry may contribute to tumor relapse and chemoresistance in NB [6]. Thus, we speculate that vasculogenic mimicry is one of the ways in which NBs sustain a blood supply independently from classical angiogenesis, which can explain, at least in part, that VEGF blockade leads only to partial response in our NB model. The most promising antiangiogenic strategies in NB will certainly include combinations of agents targeting several mechanisms of tumor angiogenesis (sprouting, co-option, vasculogenic mimicry and vasculogenesis) and directly targeting tumor cells.

## 4. Materials and Methods

### 4.1. Patients

The study has been approved by the Ethics Committee of the Medical University of Vienna (100/2000, 13 March 2000). Biopsies and serum samples were obtained from 37 patients with NB, following a written informed consent given by the parents of the children. Patients were scheduled for surgical intervention and had routine medical programs before surgery. As controls, adrenal biopsies and serum were obtained from seven children diagnosed with congenital ureteropelvic junction obstruction who underwent Anderson-Hynes pyeloplasty, following a written informed consent. All biopsies were analyzed by histological screening according to standard procedures. Tumor and adrenal biopsies, as well as serum of patients and controls, were analyzed using qRT-PCR, Western blotting, and enzyme-linked immunosorbent assay (ELISA) as described below.

### 4.2. Cell Lines

Human NB cell line, SK-N-AS (*MYCN* single copy, chromosomal status of 1p36: loss of heterozygosity (LOH), derived from a six year old girl with a poorly-differentiated NB) [44] and human embryonic kidney 293 cells, HEK293, were purchased from the American Type Culture Collection (CRL-1573; ATCC, Manassas, VA, USA). SK-N-AS cells were maintained in Dulbecco’s modified Eagle’s medium (DMEM, Life Technologies, Carlsbad, CA, USA) supplemented with 0.1 mM non-essential amino acids (NEAA, Life Technologies) and fetal calf serum to a final concentration of 10% fetal bovine serum (FBS, Life Technologies). HEK293 cells were cultured in Eagle’s minimum essential medium (EMEM, Life Technologies) supplemented with 10% FBS. Cells were cultured at 37 °C under 5% CO_2_. Cell lines were tested for authenticity by using STR-PCR (PowerPlex 16 HS System, Promega, Madison, WI, USA).

### 4.3. Generation of PlGF- and VEGF-Specific shRNA-Expressing Ads

To generate functional recombinant adenovirus constructs for silencing of human and mouse PlGF or VEGF-A, shRNAs with *BamHI* and *EcoRI* restriction site overhangs were designed. The target sequences of the used shRNAs were: hu/muPlGF: 5′-CCAAGCCAGATTCTCTTGA-3′ and hu/muVEGF: 5′-CCTCCGAAACCATGAACTT-3′ The sequences of the used shRNAs oligonucleotides were: hu/muPlgf-sh-s: 5′-GATCCGCCAAGCCAGATTCTCTTGATTCAAGAGATCAAGAGAATCTGGCTTGGTTTTTTACGCGTG-3′, hu/muPlgf-sh-as: 5′-AATTCACGCGTAAAAAACCAAGCCAGATTCTCTTGATCTCTTGAATCAAGAGAATCTGGCTTGGCG-3′; hu/muVEGF-sh-s: 5′-GATCCGCCTCCGAAACCATGAACTTTTCAAGAGAAAGTTCATGGTTTCGGAGGTTTTTTACGCGTG-3′, hu/muVEGF-sh-as: 5′-AATTCACGCGTAAAAAACCTCCGAAACCATGAACTTTCTCTTGAAAAGTTCATGGTTTCGGAGGCG-3′. shPlGF and shVEGF genes were first subcloned into RNAi-Ready pSIREN shuttle vector (Clontech, Mountain View, CA, USA) using *BamHI*-*EcoRI*, generating pSIREN-shPlGF and pSIREN-shVEGF. The shRNA sequences were screened for functionality in mouse S3T3 fibroblasts (ATCC) and SK-N-AS cells. The shRNA expression cassette from the newly-constructed pSIREN-shPlGF and pSIREN-shVEGF shuttle vectors was then ligated into the replication-deficient Ad5 genome-based 32kb BD Adeno-X Viral DNA (Adeno-X expression System 1, Clontech) using *Pi-SceI*/*I-CeuI*. The resulting Adeno-X-shPlGF and -VEGF product was then transformed into *Escherichia coli* for expansion and purified using NucleoBond Plasmid Midi Kit (Clontech). HEK293 cells were then transfected with *PacI*-digested Adeno-X constructs using Lipofectamine (Life Technologies). The viruses were propagated in HEK293 cells and purified using the Adeno-X Virus Purification kit (Clontech) according to the manufacturers’ protocol. The adenoviral titers were determined using the Adeno-X Rapid Titer kit (Clontech). The function of viruses was evaluated in SK-N-AS cells. SK-N-AS cells were infected with a multiplicity of 100 pfu/cell in six-well plates and the down-regulation of the target genes was determined by qRT-PCR and Western blotting.

### 4.4. Construction of the Ad(s)VEGFR2 Adenoviral Vector

The soluble murine VEGFR2 open reading frame was purchased from Invivogen (San Diego, CA, USA) and amplified with forward (5′-GGAATTCCACCATGGAGAGCAAGGCGCTGC-3′) and reverse (5′-CGGGATCCCGCTAGGCACCTTCTATTATGA-3′) primers incorporating *EcoRI* and *BamHI* linkers. The 2289 bp construct was gel purified with Qiaex II (Qiagen, Valencia, CA, USA) and recombinant adenovirus Ad(s)VEGFR2 was constructed as described [45]. Adeno-X-DSRed2 adenovirus for expressing red fluorescent protein (AdRFP) was obtained from Clontech and propogated in HEK293 culture. Viruses were purified with Adeno-X Maxi Purification kit (Clontech), and the infectious units were determined by hexon antibody staining of HEK293 infections (Adeno-X Rapid Titer Kit; Clontech). The functional viruses were evaluated in SK-N-AS cells by flow cytometry, immunohistochemistry, and Western blotting.

### 4.5. Quantitative Real-Time RT-PCR (qRT-PCR)

NB cells and tissues were processed for qRT-PCR as described [46]. The primer sequences for human and mouse factors (sense/antisense) are shown in Table 2. The specificity of the human and mouse primers was tested by examining melting curves of the products obtained by using both human- and mouse-specific primers on RNA from human neuroblastoma cells, mouse macrophages, and fibroblasts as described [47].

LCDA Version 3.5.3 (Roche, Mannheim, Germany) was used for PCR data analysis. Relative quantification of the signals was performed by normalizing the signals of the different genes to β2-microglobulin as described [46].

### 4.6. Protein Isolation and Western Blotting

NB cells and tissues were processed for Western blotting as described [48]. In brief, cell lysates of NB tissues and control tissues were blotted on a nitrocellulose membrane and PlGF-1 and -2 protein expression were detected with a rabbit polyclonal antibody against PlGF (Abcam, Cambridge, UK) and VEGF-A protein expression was detected with a rabbit polyclonal antibody against VEGF-A (Santa Cruz Biotechnology, Santa Cruz, CA, USA), before incubation with horseradish peroxidase-conjugated secondary antibody (Amersham Pharmacia Biotech, Buckinghamshire, UK). To measure the secretion of (s)VEGFR2 in SK-N-AS cells following infection compared to RFP controls, supernatants from Ad(s)VEGFR2 and AdRFP infected HEK293 cells were loaded on 7.5% acrylamide gels and semi-dry-blotted on nitrocellulose membranes and (s)VEGFR2 was detected with goat anti-mouse VEGFR2/FLK-1 antibody (LifeSpan BioSciences, Seattle, WA, USA) followed by a donkey anti-goat HRP conjugated IgG secondary antibody (LifeSpan BioSciences). Proteins were immunodetected by chemiluminescence (Ace Glow, Peqlab, Erlangen, Germany), scanned using FUSION-FX7 (Vilber Lourmat, Marne-la-Vallée, France) and quantified by Fusion-CAPT-Software 16.07 (Vilber Lourmat).

### 4.7. Enzyme-Linked Immunosorbent Assay (ELISA)

Immunoassay (ELISA) for human PlGF (Quantikine, R&D Systems, Minneapolis, MN, USA) was performed using 100 µL of cell culture supernatant according to manufacturer’s protocol. The absorbance was detected using a microplate reader (Thermo Fisher Scientific, Waltham, MA, USA) at 450 nm.

### 4.8. Fluorescence-Activated Cell Sorting (FACS) Analysis

SK-N-AS cells were seeded in 10 cm plates and allowed to adhere before infection with Ad(s)VEGFR2 and one portion of the cells were left untreated. Cells were washed with PBS and then fixed with Cytofix fixation buffer (BD Biosciences, San Diego, CA, USA) for 30 min at 37 °C, washed, and then permeabilized with Perm buffer III (BD Biosciences) and stained with VEGFR2 antibody (LifeSpan BioSciences) to detect intracellular VEGFR2 expression. The analysis of 10^4^ events was conducted on a FACScan flow cytometer (BD Biosciences) with an argon laser tuned to 488 nm.

### 4.9. SK-N-AS Tumor Cell Implantation and Adenovirus Injection/Analysis of Adenoviruses in Vivo

The experiments performed in this study were approved by the Institutional Animal Care and Use Committee at the Medical University of Vienna. Pathogen free immune-deficient male athymic nu/nu (nude) mice (Charles River, Sulzfeld, Germany), five weeks of age were weighed, coded, and randomly assigned to experimental groups of *n* = 4. Mice were anesthetized (ketamine hydrochloride/xylazine at 55/7.5 mg/kg, intraperitoneally, and 4 × 10^6^ SK-N-AS cells/150 μL PBS were injected subcutaneously into their left flank. In the present study, mice developed human NBs of similar weight at 10 days. Treatment was started on day 10 at a dose of 10^9^ Ad(sh)PlGF, Ad(sh)VEGF, or Ad(s)VEGFR2 viruses in 100 µL PBS intratumorally. Control animals received injections of 10^9^ AdRFP in PBS or 100 µL PBS only. Treatment was repeated twice at five day intervals. Tumor mass was determined on termination. All mice were sacrificed on day 22, and tumors were isolated and weighed. One portion of the tissue was processed for paraffin embedding, and the remainder was processed for qRT-PCR.

### 4.10. Histology and Immunohistochemistry

Cells were harvested from AdRFP and Ad(s)VEGFR2 infected SK-N-AS cultures. Cytospins were fixed in acetone for 8 min at 4 °C, and sequentially incubated with primary goat anti-mouse VEGFR2/FLK-1 antibody (LifeSpan BioSciences), a biotinylated donkey anti-goat IgG secondary antibody (LifeSpan BioSciences), and horseradish peroxidase (HRP)-conjugated streptavidin (Dako, Glostrup, Denmark) (all for 30 min at room temperature) developed with 3,3′-diaminobenzidine (Vector Laboratories, Burlingame, CA, USA), counterstained with hemalaun, dehydrated, and mounted in DPX (distyrene, plasticizer, xylene; Merck, Darmstadt, Germany).

Paraffin-embedded tumor sections were rehydrated, incubated in 5% H_2_O_2_ to block endogenous peroxidase activity and antigens were stained with hematoxylin and eosin (H and E), or subjected to immunohistochemical analysis for Ki-67 antibody (tumor proliferation assay; Dako, Glostrup, Denmark) to evaluate the density of proliferating cells [49], and anti-CD34 antibody (Rabbit monoclonal (EP373Y) to CD34, Abcam) to evaluate the density of endothelial cells. Primary antibodies were detected with biotinylated secondary antibody (Vector Laboratories) and peroxidase conjugated streptavidin (Dako), developed with 3,3′-diaminobenzidine (Vector Laboratories), counterstained with hemalaun, dehydrated and mounted in DPX (Merck). Digitalized images were generated with a Nikon Eclipse 80i (Tokyo, Japan) microscope and analyzed using NIS Elements imaging software (Nikon). Results are expressed as number of Ki-67-positive cells per high-power field (400× magnification). Endothelial microvessel density was assessed by anti-CD34 staining and morphological analysis and examination of 10–20 microscopic fields per tumor. Tumor sections were scanned at low magnification (20× objective), and tumor areas with high microvessel density (hot-spots) were selected. Microvessels were counted in representative high magnification (40× objective) fields in each area. Hemorrhagic and necrotic areas were avoided. Endothelial microvessels with a clearly defined lumen or well-defined linear vessel shape were taken into account for microvessel counting.

### 4.11. Statistical Analysis

Differences between groups were studied using analysis of variance (ANOVA) followed by post hoc tests (Dunnet, Tukey). All statistical tests were two-sided. Data are expressed as means ± the standard deviation (SD). *p* values of <0.05 were considered to indicate statistical significance. Statistical tests were performed with the use of Statistical Package for the Social Sciences (SPSS) software (version 22.0, SPSS Inc., Chicago, IL, USA).

## 5. Conclusions

In this study, we show that PlGF is up-regulated in NB tissue and serum of NB patients and that PlGF acts as a positive regulator of NB growth. Moreover, we identified PlGF as a potential therapeutic target in high-risk *MYCN*-non-amplified NB. PlGF inhibition by virally-expressed shPlGF reduced tumor growth in a *MYCN*-non-amplified NB xenograft model by affecting proliferation of tumor cells without remodeling of the vascular network. In contrast, blockade of VEGF-A by using virally-expressed shVEGF-A or sVEGFR2, which antagonizes VEGFR2 activity, reduced tumor growth associated with vessel normalization and decreased hemorrhage. A multiple target approach based on the combination of PlGF blockade with anti-tumor cell activity and anti-vascular therapies could be expected to improve the therapeutic effects of anti-angiogenic strategies against NB.

## Figures and Tables

**Table 1:** Patient characteristics.

Group	Age, Months	Sex, M/F	Tumor Location
Control	21 ± 17	3/4	–
Stage I *	7 ± 4	5/3	Adrenal
Stage II	9 ± 3	7/3	9 Adrenal/1 Vagus nerve
Stage III	10 ± 4	4/5	8 Adrenal/1 Sympathetic trunk
Stage IV	14 ± 11	6/4	Adrenal

* Staging according to Evans. M, male; F, female.

**Table 2:** Primer sequences (sense/antisense).

Gene	Primer Sense 5′–3′	Primer Antisense 5′–3′
human PlGF	GTTCAGCCCATCCTGTGTCT	CTTCATCTTCTCCCGCAGAG
human VEGF-A	AGCCTTGCCTTGCTGCTCTA	GTGCTGGCCTTGGTGAGG
human FLT-1	GTCACAGAAGAGGATGAAGGTGTC	CACAGTCCGGCACGTAGGTGATT
human KDR	GCATCTCATCTGTTACAGC	CTTCATCAATCTTTACCCC
mouse PlGF	GAGCTTCGGCTTGGGAAGAAG	GTTCCAGAGAGGGGACAAAGG
mouse VEGF-A	GCAGAAGTCCCATGAAGTGATC	CTCCAGGGCTTCATCGTTACAG
mouse FLT-1	AAACCTGTCCAACTACCTC	TAATCCTCGTCTCCTTCC
mouse KDR	GGAGATTGAAAGAAGGAAC	ACTTCCTCTTCCTCCATAC

PlGF, placental growth factor; VEGF-A, vascular endothelial growth factor A; FLT-1, fms related tyrosine kinase 1; KDR, kinase insert domain receptor.
